# Retention of subcutaneous abatacept for the treatment of rheumatoid arthritis: real-world results from the ASCORE study: an international 2-year observational study

**DOI:** 10.1007/s10067-022-06176-1

**Published:** 2022-05-10

**Authors:** Rieke Alten, Xavier Mariette, René-Marc Flipo, Roberto Caporali, Maya H. Buch, Yusuf Patel, Sara Marsal, Raimon Sanmartí, Michael T. Nurmohamed, Hedley Griffiths, Peter Peichl, Bettina Bannert, Melanie Chartier, Sean E. Connolly, Karissa Lozenski, Christiane Rauch

**Affiliations:** 1 Schlosspark-Klinik, University Medicine Berlin, Heubnerweg 2, 14059 Berlin, Germany; 2 Université Paris-Saclay, AP-HP, Hospital Bicêtre, INSERM UMR1184, Le Kremlin Bicêtre, France; 3 Centre Hospitalier Universitaire de Lille, Lille, France; 4 University of Milan, Milan, Italy; 5 G. Pini Hospital, Milan, Italy; 6 University of Leeds, Leeds, UK; 7 University of Manchester and NIHR Manchester Biomedical Research Centre, Manchester, UK; 8 Hull University Teaching Hospitals NHS Trust, Hull, UK; 9 Hospital Universitari Vall d’Hebron, Barcelona, Spain; 10 Hospital Clínic de Barcelona, Barcelona, Spain; 11 ARC Amsterdam University Hospitals – VU University Medical & Reade, Amsterdam, Netherlands; 12 University Hospital Geelong, Geelong, VIC Australia; 13 Evangelical Hospital, Vienna, Austria; 14 Universitätsspital Basel, Basel, Switzerland; 15 Bristol Myers Squibb, Rueil-Malmaison, France; 16 Bristol Myers Squibb, Princeton, NJ USA; 17 Bristol Myers Squibb, Munich, Germany

**Keywords:** Abatacept, Biological therapy, Retention, Rheumatoid arthritis

## Abstract

**Objectives:**

To evaluate retention, efficacy, and safety of subcutaneous (SC) abatacept over 2 years in patients with moderate-to-severe RA in the Abatacept SubCutaneOus in Routine clinical practicE (ASCORE) study.

**Methods:**

Patients with RA who initiated SC abatacept 125 mg once weekly were enrolled in the international, observational, prospective multicentre ASCORE study into biologic-naïve or ≥ 1 prior biologic failure cohorts. Primary endpoint: abatacept retention rate at 2 years. Secondary endpoints: proportion of patients with good/moderate EULAR response rates based on DAS28 (ESR), low disease activity and/or remission according to DAS28 (ESR; ≤ 3.2/ < 2.6), SDAI (≤ 11/ ≤ 3.3), CDAI (≤ 10/ ≤ 2.8), and Boolean criteria. Retention rate by baseline serostatus was evaluated post hoc.

**Results:**

Overall, 47% of patients remained on abatacept for 2 years, irrespective of treatment line. Higher abatacept retention rates were associated with lower prior biologic exposure. Generally, clinical outcomes showed that the proportion of patients with low disease activity/remission was higher in biologic-naïve patients (vs biologic-failure) and similar in those with 1 and ≥ 2 prior biologic failures. In patients on treatment at 2 years, good/moderate EULAR response rates of ~ 80% were consistently noted irrespective of prior biologic exposure. Across treatment lines, retention was greater in patients with seropositive (vs seronegative) RA. Patients with rheumatoid factor/anti-citrullinated protein antibody single-positive RA who were bio-naïve had higher retention than patients who were bio-experienced.

**Conclusions:**

In the ASCORE study, SC abatacept retention was 47% at 2 years with good clinical outcomes and was well-tolerated in the real-world setting. Abatacept retention and clinical response rates were higher in patients who received abatacept as an earlier- versus later-line biologic drug treatment and in those with seropositive RA.

**Trial registration:**

ClinicalTrials.gov, NCT02090556.

**Supplementary Information:**

The online version contains supplementary material available at 10.1007/s10067-022-06176-1.

## Introduction

Rheumatoid arthritis (RA) is a chronic autoimmune disease characterised by systemic inflammation that leads to structural joint damage in nearly all patients, if left untreated [1]. The presence of rheumatoid factor (RF) and anti-citrullinated protein antibodies (ACPAs) are associated with a severe and aggressive disease course in patients with RA [2] and the effect of serostatus on response to therapy is not fully understood. For patients with established RA, treatment approaches aim for sustained clinical remission, with low disease activity (LDA) as a possible alternative outcome [3, 4]. The treat-to-target approach is advocated both by the American College of Rheumatology (ACR) and the European League Against Rheumatism (EULAR), and early use of immunomodulatory biologic and targeted synthetic disease-modifying antirheumatic drugs (bDMARDs/tsDMARDs) when conventional synthetic DMARDs (csDMARDs) fail to reach therapeutic targets by 3 to 6 months is recommended [3, 4].

Abatacept is a selective co-stimulation modulator that blocks the interaction between CD80/CD86 on antigen-presenting cells and CD28 on T cells, thus disrupting the continuous cycle of T cell activation [5]. Abatacept has proven efficacy and safety in the treatment of patients with RA and, along with other bDMARDs, it is approved for the treatment of moderate-to-severe RA [6–8]. The SC and IV formulations of abatacept have demonstrated comparable efficacy (ACR 20% improvement criteria [ACR20] response was met by 76.0% of SC and 75.8% of IV abatacept-treated patients) and safety in the phase III ACQUIRE (Abatacept Comparison of Sub[QU]cutaneous versus Intravenous in inadequate REsponders to methotrexate) study [6]. Additionally, ACPA positivity has been associated with a better response with and greater retention of abatacept than ACPA negativity [9, 10].

Stringent requirements for participation in randomised controlled trials (RCTs) means that study participants may not fully represent the clinical population [11]. As such, real-world data provide valuable insights into the long-term use of bDMARDs in patients with RA in clinical settings [12].

ASCORE (Abatacept SubCutaneOus in Routine clinical practicE) was a 2-year, observational, prospective multicentre study of the efficacy and safety of SC abatacept for the treatment of patients with moderate-to-severe active RA in routine clinical practice. Interim analyses of the ASCORE study at 6 months and 1 year showed better retention rates and clinical response rates in patients receiving SC abatacept as a first- versus later-line bDMARD [13, 14].

The objectives of this analysis were to investigate the patient treatment retention, efficacy, and safety of SC abatacept in routine clinical practice by previous biologic exposure. Here we report the final 2-year results for all patients enrolled in the ASCORE study.

## Methods

### Study design

ASCORE was an international, observational, prospective multicentre cohort study (ClinicalTrials.gov: NCT02090556) of patients with RA in routine clinical practice.

Patients were recruited from February 2013 until April 2017 from 10 countries across Europe (including Austria, France, Monaco, Germany, Greece, Italy, Netherlands, Spain, Switzerland, and UK) and Australia. All participating countries involved were required to have regulatory approval and market availability of the delivery device to ensure drug availability for all eligible patients. SC abatacept was initiated under the guidance of the treating physician and in accordance with local routine clinical practices. No product was provided to the physicians or patients directly by the study sponsor, and the observational design of the study did not interfere with usual local clinical practice. Patients who met inclusion criteria received follow-up approximately every 3 months for 30 months, in line with routine clinical practice. Those patients who discontinued SC abatacept, regardless of the reason and time of discontinuation, were followed up to the planned 24-month follow-up.

The rheumatologists involved in the study were randomly selected from country-specific nationwide independent databases of specialists located in hospitals or private practice for a well-balanced geographic distribution and were representative of specialists caring for patients with RA in each participating country.

The study was conducted in accordance with the Declaration of Helsinki [15], the International Conference on Harmonisation Good Clinical Practice Guidelines [16], and the International Society for Pharmacoepidemiology (ISPE) Guidelines for Good Epidemiology Practices [17]. The laws and regulatory requirements of all countries participating in this study were adhered to. The study protocol and patient enrolment materials were approved according to local law in each participating country prior to initiation of the study. All enrolled patients provided informed consent in accordance with local laws.

### Study population

Patients aged ≥ 18 years with an active moderate-to-severe RA diagnosis (as defined by ACR/EULAR 2010 criteria [18]) who were IV abatacept-naïve were eligible for inclusion in ASCORE. Patients who initiated SC abatacept 125 mg once weekly were enrolled into two cohorts: biologic-naïve patients and those with ≥ 1 failure of prior biologic treatment. Specific exclusion criteria were not defined in this observation study; however, patients who were currently participating in any interventional clinical trial in RA were excluded.

### Study assessments and outcomes

Patient demographics and disease characteristics at the time of initiation of abatacept were recorded by treatment line. Past and present comorbidities were noted.

#### Retention and efficacy

The primary endpoint of the ASCORE study was the retention rate of abatacept (defined as consecutive time on treatment over 2 years) by treatment line. Discontinuation of abatacept in this study was defined as the switch from abatacept (SC or IV formulation) to any other RA DMARD or no treatment. For the primary endpoint, patients who switched from SC to the IV formulation of abatacept were considered to have discontinued. Patients lost to 2-year follow-up were excluded from analysis.

Secondary endpoints included the proportion of patients with good/moderate EULAR response rates based on Disease Activity Score in 28 joints (DAS28; erythrocyte sedimentation rate [ESR] or C-reactive protein [CRP]) by treatment line. LDA and/or remission according to DAS28 (ESR; ≤ 3.2/ < 2.6), Simplified Disease Activity Index (SDAI; ≤ 11/ ≤ 3.3), Clinical Disease Activity Index (CDAI; ≤ 10/ ≤ 2.8), and Boolean criteria by treatment line were also assessed [19]. A post hoc exploratory endpoint was the retention rate of abatacept by baseline serostatus, RF/ACPA double-positive RA, RF/ACPA single-positive (RF + /ACPA − or RF − /ACPA +) RA, and RF/ACPA double-negative RA. Additional exploratory evaluations included change from baseline in disease activity and the proportion of patients with SDAI and CDAI LDA and/or remission in patients with RF/ACPA double-positive RA and RF/ACPA double-negative RA.

#### Safety

Safety was monitored and evaluated in accordance with local regulations. The drug manufacturer’s pharmacovigilance department was notified of any adverse events (AEs) or serious adverse events (SAEs) assessed by the treating physician whether or not related to abatacept. An SAE was defined as an AE that was fatal or life-threatening, required extended hospitalisation, led to persistent or significant disability or incapacity, induced a birth defect, or was considered an important medical event. Deaths from any cause were reported.

### Statistical analyses

The calculation of the sample size was based on accuracy of the estimation of studied endpoints. Accuracy was estimated through estimate precision (95% confidence interval [CI]) and sample size was dependent on assumption of retention rate at 24 months. At the country level, a 95% CI for an assumed 60% retention rate at 24 months > 7.8% (sample size lower than 150 in single cohort) was considered a low precision level. As a consequence, country-specific results were considered statistically relevant only for samples including a minimum of 150 patients.

For each cohort, all patients fulfilling the study selection criteria and those who received at least one dose of abatacept SC were included in the analysis population. Baseline demographics and disease characteristics were analysed descriptively and reported as mean (standard deviation [SD]) for continuous variables and *n* (%) for categorical variables. The primary endpoint, 2-year retention rate, was estimated by Kaplan–Meier analysis with 95% CI and a log-rank test for comparison of patients stratified by previous biologic treatment exposure. Clinical outcomes at 2 years were reported as percentages with 95% CI. An exploratory analysis, similar to the primary analysis, included patients who discontinued SC and switched to the IV formulation of abatacept. For the post hoc analysis, estimates of mean difference with 95% CIs between patients with different serostatus were calculated using a *t*-test by treatment line. Last observation carried forward efficacy analyses were used to impute missing values for exploratory endpoints. The statistical analyses were performed using SAS version 9.2 (SAS Institute, NC, USA). Safety was analysed descriptively throughout the study and is presented for the overall population as well as by treatment line.

## Results

### Patient disposition and baseline characteristics

**Fig. 1:**
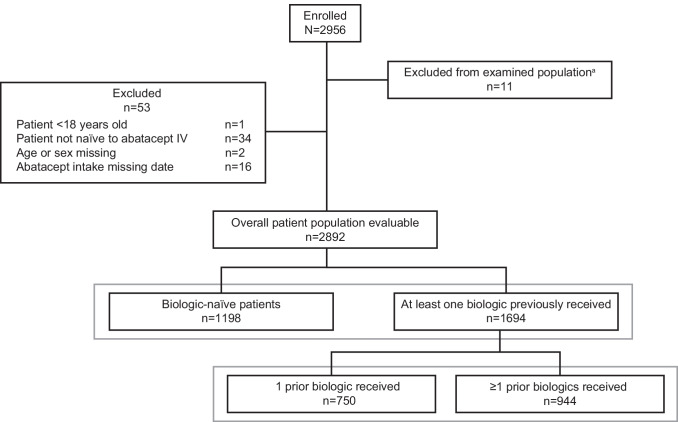
Patient disposition. ^a^Patients with no recorded date of consent or date of visit 1. IV, intravenous

In total, 2956 patients were enrolled in the ASCORE study (Fig. 1). Eleven patients were excluded from the examined population (due to no recorded date of consent or date of visit 1), leaving 2945 patients from 10 countries enrolled by 574 participating rheumatologists (online supplemental Table 1). The overall evaluable population consisted of 2892 patients (53 patients were excluded due to not meeting inclusion criteria, including 34 who were not IV abatacept-naïve): 1198 (41.4%) were biologic-naïve and 1694 (58.6%) experienced prior biologic treatment failure (Fig. 1). Of those who had prior biologic treatment failure, 750 and 944 had failed 1 and ≥ 2 treatments, respectively. Lack of efficacy (66.3%) and intolerance/safety (26.6%) were the most common reasons for discontinuation of the last previous biologic for patients with ≥ 1 failure of prior biologic treatment.

**Table 1:** Baseline patient demographics and disease characteristics

Characteristic	Biologic-naïve *n* = 1198	1 prior biologic *n* = 750	≥ 2 prior biologics *n* = 944
Age, years	57.9 (13.0)	57.6 (12.6)	57.5 (12.4)
Female, *n* (%)	914 (76.3)	597 (79.6)	761 (80.6)
BMI, kg/m^2^, *n* (%)			
< 25	466 (41.1)	277 (39.2)	337 (38.6)
25– < 30	423 (37.3)	237 (33.5)	293 (33.5)
≥ 30– < 35	150 (13.2)	121 (17.1)	149 (17.0)
≥ 35	96 (8.5) (*n* = 1135)	72 (10.2) (*n* = 707)	95 (10.9) (*n* = 874)
Presence of co-morbidity (past and present), *n* (%)	921 (76.9)	581 (77.5)	735 (77.9)
RA disease duration, years	8.2 (8.4) (*n* = 1188)	12.1 (9.5) (*n* = 746)	14.6 (9.7) (*n* = 938)
RA duration, years, *n* (%)			
≤ 2	275 (23.1)	47 (6.3)	16 (1.7)
3–5	358 (30.1)	176 (23.6)	121 (12.9)
6–10	244 (20.5)	188 (25.2)	254 (27.1)
> 10	311 (26.2) (*n* = 1188)	335 (44.9) (*n* = 746)	547 (58.3) (*n* = 938)
Presence of radiographic erosion, *n* (%)	422 (48.1) (*n* = 877)	282 (55.0) (*n* = 513)	413 (64.3) (*n* = 642)
ACPA positive, *n* (%)	632 (74.9) (*n* = 844)	342 (70.2) (*n* = 487)	380 (69.0) (*n* = 551)
RF positive, *n* (%)	659 (71.9) (*n* = 916)	368 (68.9) (*n* = 534)	426 (67.2) (*n* = 634)
DAS28 (CRP)	4.7 (1.1) (*n* = 887)	4.7 (1.2) (*n* = 514)	4.7 (1.2) (*n* = 600)
CRP, mg/dL	16.0 (28.4)	15.9 (34.4)	15.0 (22.1)
ESR, mm/h	30 (23.4)	29.7 (23.6)	28.2 (24.7)
Tender joint count (28 joints)	8.9 (6.6)	8.6 (6.8)	9.0 (6.8)
Swollen joint count (28 joints)	6.3 (5.1)	5.9 (5.3)	6.2 (5.4)
HAQ-DI	1.31 (0.73)	1.35 (0.70)	1.53 (0.72)
Pain^a^	57.0 (22.5)	60.3 (22.3)	60.7 (22.6)
Prior treatment with MTX, *n* (%)	1086 (90.7)	651 (86.8)	754 (79.9)
Prior treatment with glucocorticoids, *n* (%)	934 (78.0)	588 (78.4)	733 (77.6)
Current treatment with glucocorticoids, *n* (%)	774 (64.6)	484 (64.5%)	635 (67.3%)
Number of previous anti-TNF			
Mean (SD)	–	1.0 (0.0)	2.6 (0.8)
1, *n* (%)	–	646 (86.1)	255 (27.0)^b^
≥ 2, *n* (%)	–	0 (0.0)	679 (71.9)
Median (range) number of TNFis	0.0 (0.0, 0.0)	1.0 (1.0, 1.0)	2.0 (1.0, 2.0)
Prior anti-TNF/bDMARD, *n* (%)			
Anti-TNF + bDMARD	–	0	530 (56.1)
Anti-TNF	–	646 (86.1)	404 (42.8)
bDMARD	–	104 (13.9)	10 (1.1)

Data are shown as mean (SD) unless otherwise specified. Overall analysis population

^a^0–100 mm visual analogue scale

^b^1 previous anti-TNF + other bDMARD

*ACPA*, anti-citrullinated protein antibody; *bDMARD*, biologic disease-modifying antirheumatic drug; *BMI*, body mass index; *CRP*, C-reactive protein; *DAS28*, Disease Activity Score in 28 joints; *ESR*, erythrocyte sedimentation rate; *HAQ-DI*, Health Assessment Questionnaire–Disability Index; *MTX*, methotrexate; *RA*, rheumatoid arthritis; *RF*, rheumatoid factor; *SD*, standard deviation; *TNF*, tumour necrosis factor

At baseline, overall patient demographics (mean age 58 years, 79% female, 77% reported comorbidities, of which hypertension, dyslipidaemia, hypothyroidism, and diabetes were most frequently reported) and disease characteristics were similar across treatment lines (Table 1). However, trends were noted between patients who had a higher versus lower number of prior biologic treatments. For example, a higher proportion of patients with ≥ 2 prior biologic treatment failures had a longer disease duration and more erosive disease compared to those with failure of 1 prior biologic treatment and patients who were biologic-naïve. In biologic-naïve patients, higher methotrexate use was noted compared to those patients with prior biologic treatment failure. At treatment initiation, biologic-naïve patients had higher concomitant methotrexate, csDMARD, and glucocorticoid use than patients with prior biologic treatment failure (data not shown). Baseline demographics and disease characteristics were similar across serostatus groups and treatment lines (online supplemental Table 2).

**Table 2:** Summary of adverse events experienced by patients overall and by treatment line

	Biologic-naïve *n* = 1198	1 prior biologic *n* = 750	≥ 2 prior biologics *n* = 944	Total *N* = 2892
Adverse event	640 (53.4)	403 (53.7)	533 (56.5)	1576 (54.5)
Serious adverse events	199 (16.6)	123 (16.4)	154 (16.3)	476 (16.5)
Site reaction^a^	18 (2.2) (*n* = 810)	11 (2.6) (*n* = 430)	12 (2.4) (*n* = 490)	41 (2.4) (*n* = 1730)
Related serious adverse events	96 (8.0)	61 (8.1)	69 (7.3)	226 (7.8)
Abatacept discontinuation due to death	6 (1.1)	3 (0.8)	3 (0.6)	12 (0.8)
Serious adverse event related to infections and infestations	53 (4.4)	28 (3.7)	26 (2.8)	107 (3.7)
Serious adverse event related to malignancy	14 (1.2)	6 (0.8)	5 (0.5)	25 (0.9)

All values are number of patients (%)

^a^In Germany and Spain, only prevalent patients could be included; therefore, to avoid bias they were excluded from this data set

### Efficacy

#### Retention

**Fig. 2:**
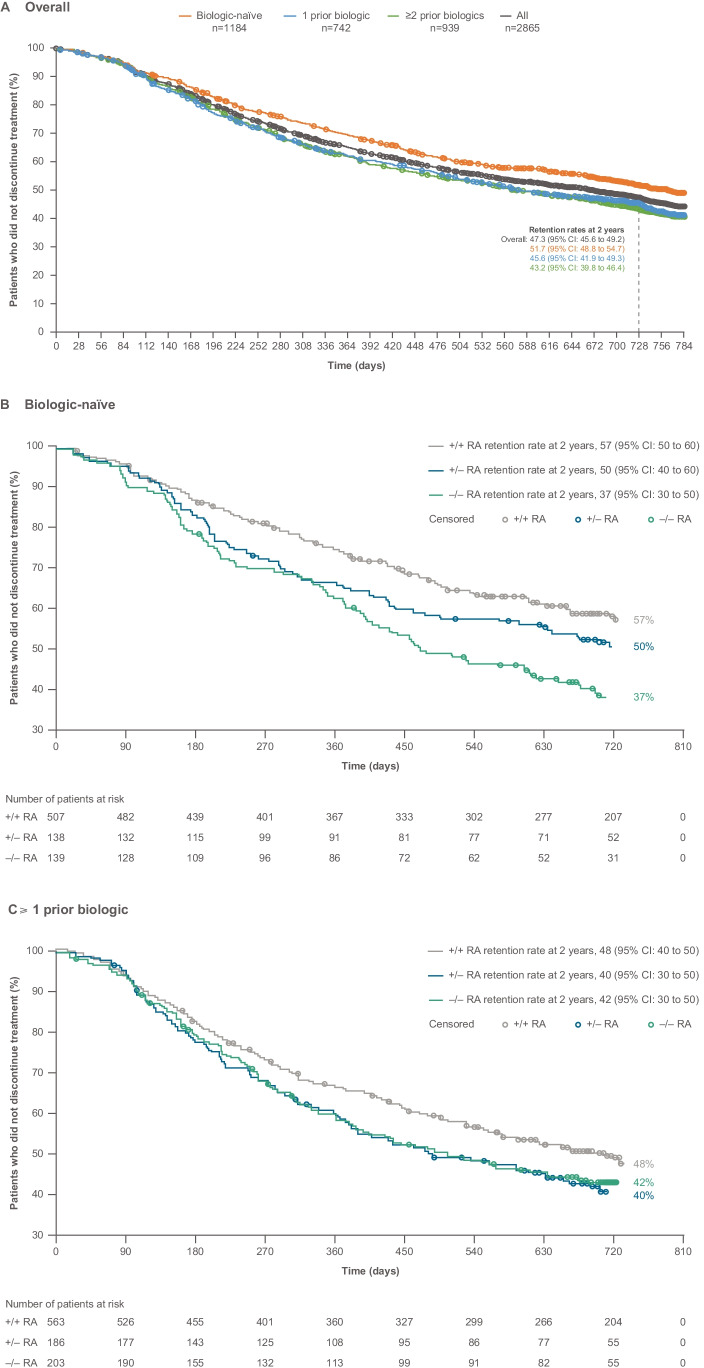
Proportion of patients with subcutaneous abatacept retention over 2 years by treatment line^a^: **A** overall, **B** biologic-naïve, and **C** ≥ 1 prior biologic failure.^b a^Patients who switched to IV abatacept during the 2 years were discontinued and are not included. ^b^First line or more data present combined data from patients with 1 and ≥ 2 failures of prior biologic treatment, as further stratification would result in small patient numbers. Panels **B** and **C** reproduced from Alten R, et al. EULAR Virtual Congress 2021; 3 June 2021; oral presentation OP0180 (with permission from the authors). CI, confidence interval; KM, Kaplan–Meier; IV, intravenous; RA, rheumatoid arthritis

Overall SC abatacept crude retention rate (95% CI) at 2 years was 47.3% (45.6 to 49.2). Retention was higher in patients receiving abatacept as a first- versus later-line biologic; 51.7% (48.8 to 54.7) in biologic-naïve patients, 45.6% (41.9 to 49.3) in those with ≥ 1 prior biologic treatment failure, and 43.2% (39.8 to 46.4) in those with ≥ 2 prior biologic treatment failures (Fig. 2A). In patients with RF/ACPA double-positive RA, abatacept retention was greater for biologic-naïve patients than in those with ≥ 1 prior biologic treatment failure (57% vs 48%) (Fig. 2B and C; combined data from patients with 1 and ≥ 2 failures of prior biologic treatment presented as further stratification would result in small patient numbers). Retention in patients with RF/ACPA single-positive RA was greater in biologic-naïve patients compared to those with ≥ 1 prior biologic treatment failure (50% vs 40%), while retention in patients with RF/ACPA double-negative RA was similar regardless of treatment line (biologic-naïve patients 37% vs 42% for those with ≥ 1 prior biologic treatment failure). Retention in patients with RF/ACPA double-positive RA was greater than that seen in patients with RF/ACPA double-negative RA across treatment lines.

At 2 years, 1459 patients had discontinued SC abatacept—the majority (*n* = 643; 44.1%) due to inefficacy, with 304 (20.8%) discontinuing due to safety, and 42 (2.9%) lost to follow-up and excluded from analysis. These discontinuations of SC abatacept remained linear throughout the study time period (data not shown).

#### Clinical outcomes and exploratory analysis

**Fig. 3:**
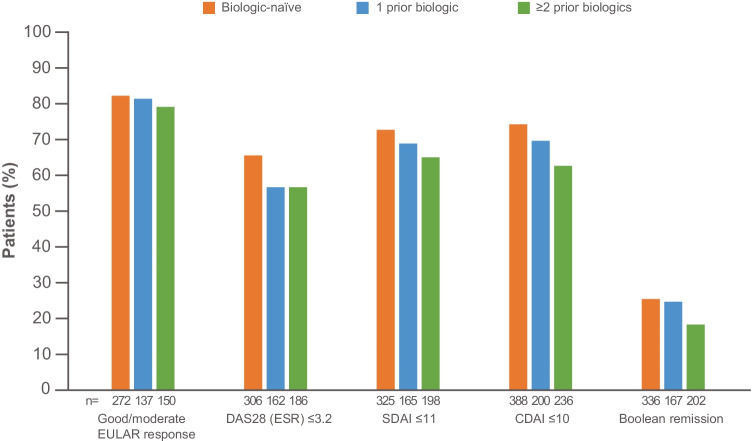
Clinical outcomes at 2 years by treatment line. DAS28, Disease Activity Score in 28 joints; CDAI, Clinical Disease Activity Index; ESR, erythrocyte sedimentation rate; EULAR, European League Against Rheumatism; SDAI, Simplified Disease Activity Index

In patients on treatment at 2 years, the proportion of patients with good/moderate EULAR response rates were similar—82.4% (224/272), 81.8% (112/137), and 79.3% (119/150) at 2 years—in biologic-naïve patients as well as in patients with 1 and ≥ 2 failures of prior biologic treatment, respectively (Fig. 3). The corresponding proportions of patients with DAS28 (ESR), SDAI, CDAI LDA and/or remission, and Boolean remission were higher with abatacept as an earlier- versus later-line biologic at 2 years (Fig. 3).

**Fig. 4:**
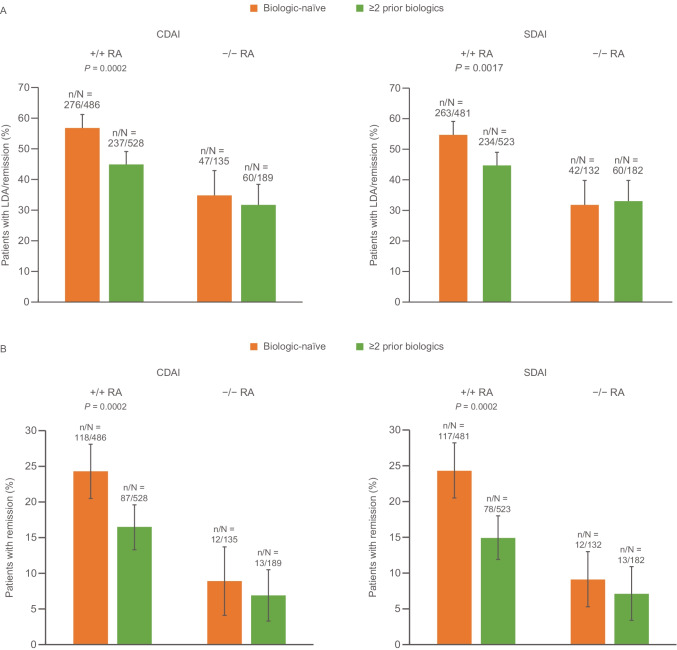
SDAI and CDAI **A** LDA/remission and **B** remission at 2 years by serostatus and treatment line (last observation carried forward). Figure reproduced from Alten R, et al. EULAR Virtual Congress 2021; 3 June 2021; oral presentation OP0180 (with permission from the authors). CDAI, Clinical Disease Activity Index; LDA, low disease activity; RA, rheumatoid arthritis; SDAI, Simplified Disease Activity Index

Exploratory evaluations of change from baseline in disease activity scores (SDAI and CDAI) at 24 months demonstrated that patients with RF/ACPA double-positive RA treated with first-line abatacept experienced a greater reduction in disease activity than those receiving later-line therapy. Patients with RF/ACPA double-negative RA experienced less reduction in disease activity regardless of therapy line (data not shown). Comparable changes from baseline were observed in DAS28 (CRP) scores (data not shown). The proportion of patients with SDAI and CDAI LDA and/or remission in patients with RF/ACPA double-positive RA and RF/ACPA double-negative RA was higher with abatacept as an earlier- versus later-line biologic at 2 years (Fig. 4A). A lower proportion of patients with RF/ACPA double-negative (compared with RF/ACPA double-positive) RA achieved LDA/remission regardless of line of treatment (Fig. 4A). The proportion of biologic-naïve patients with CDAI and SDAI remission was 24.3% and 24.3% (RF/ACPA double-positive RA) and 8.9% and 9.1% (RF/ACPA double-negative RA), respectively (Fig. 4B).

In an exploratory analysis including those patients (*n* = 7; 0.5%) who discontinued SC and switched to the IV formulation of abatacept, overall retention at 2 years (47.9 [95% CI 46.0 to 49.8]) was similar to the main analysis where these patients were excluded (online supplemental Fig. 1).

### Safety

Safety profiles were similar across treatment lines, with no new signals for abatacept reported during the study period. Overall, 54.5% of patients reported ≥ 1 AE (1576/2892) and 16.5% of patients reported ≥ 1 SAE (476/2892), with 226 (7.8%) patients having SAEs related to abatacept (Table 2). There were 35 deaths during the study period, and abatacept discontinuation due to death was recorded for 12 patients (Table 2).

## Discussion

The findings from the prospective international ASCORE study of SC abatacept in real-world clinical practice showed that overall, 47% of patients remained on abatacept treatment for 2 years. Higher retention of abatacept at 2 years was associated with lower previous exposure to biologics, with biologic-naïve patients showing a retention rate of 52% compared to a rate of 46% in patients with previous prior biologic treatment failure. In this real-world analysis, biologic-naive patients with RF and ACPA positive RA also had higher abatacept retention than those with previous prior biologic treatment failure. In addition, patients with RF/ACPA double-positive RA had greater retention greater than those with RF/ACPA double-negative RA.

Clinical outcomes showed that the proportion of patients with DAS28 (ESR) ≤ 3.2 was higher in patients who were biologic naïve than in those with biologic failure and similar in those with 1 and ≥ 2 failures of prior biologic treatment. The proportion of patients in SDAI and CDAI LDA and/or remission were most improved in the biologic-naïve cohort. The proportion of patients in Boolean remission was similar in the biologic-naïve and 1 prior biologic treatment failure lines and higher than in the ≥ 2 prior biologic treatments failure line. In patients on treatment at 2 years, good/moderate EULAR response rates were consistently noted in approximately 80% of patients irrespective of prior biologic exposure. These observations are in line with the interim analyses of the ASCORE study that showed better retention rates (6 months, 88%; 1 year, 65%) and clinical response rates in patients receiving SC abatacept as a first- versus later-line bDMARD [13, 14]. Exploratory evaluations of clinical outcomes by serostatus showed greater reduction in disease activity (SDAI and CDAI) in patients with RF/ACPA double-positive RA treated with first-line abatacept than in those receiving later-line therapy and those who had RF/ACPA double-negative RA. In addition, a higher proportion of patients with RF/ACPA double-positive RA were in SDAI and CDAI LDA and/or remission compared with patients with RF/ACPA double-negative RA. Similarly, a higher proportion of patients who received earlier-line abatacept were in SDAI and CDAI LDA and/or remission compared with patients who received abatacept as a later-line biologic with RF/ACPA double-negative RA. The higher retention and responder rates seen in patients with seropositive disease are consistent with abatacept’s mechanism of action of interrupting T-cell co-stimulation and associated downstream processes, such as ACPA generation, by binding CD80/CD86 on B cells [20]. These data support the importance of precision medicine in treating patients with RA.

The retention rates reported here were consistent with findings from independent registry studies for SC and IV abatacept [21–23]. Similar to those seen with the SC formulation, IV abatacept retention rates overall in the real-world AbataCepT In rOutiNe clinical practice (ACTION; recruited patients 2008–2013) study were comparable to those reported here; for ACTION at 2 years, overall retention was 48% [24]. In addition, retention rates reported in the ACTION study in earlier lines of treatment were higher than those in later lines of treatment in patients with RA; these being 55% for biologic-naïve and 45% for biologic-failure patients [10, 25, 26]. A 2-year abatacept retention rate of 39.3% was reported for patients with biologic failure in the Orencia and Rheumatoid Arthritis (ORA) registry [23]. An observational study, based on data from the French-RIC Network, reported a 2-year abatacept persistence of 52% in patients with RA [27]. In RCTs, higher retention rates of abatacept are reported; for example, in excess of 80% of patients remained in the Abatacept Comparison of sub[QU]taneous versus intravenous in Inadequate Responders to methotrexatE (ACQUIRE) study (up to a maximum of 3.5 years) [28] and at year 2 of the Abatacept or infliximab versus placebo, a Trial for Tolerability, Efficacy and Safety in Treating rheumatoid arthritis (ATTEST) study [29] and at year 1 of the Abatacept versus adaliMumab comParison in bioLogic-naïvE rheumatoid arthritis subjects with background methotrexate (AMPLE) studies [30]. These differences may be in part due to the variations in patient populations, particularly with RCT participants not being fully representative of the clinical population [11].

The retention rates reported with other biologics are generally similar to those reported in the current analysis for abatacept (online supplemental table S3) [31–35]. In the clinical practice setting, a 61% retention rate was observed at 2 years for tocilizumab [35], whereas a registry study reported 3-year tocilizumab retention rates as 52% in bDMARD-naïve patients and 51% and 47% in patients with 1 or 2 prior bDMARD failures, respectively [32]. A population-based prospective study in France reported 2-year retention rates of 69% for rituximab, 39% for abatacept, and 63% for tocilizumab in patients with RA in routine practice [23]. In addition, 2-year retention rates of 31%, 40%, and 53% have been noted for adalimumab, etanercept, and golimumab, respectively, in an Italian registry study of patients with RA who previously failed tumour necrosis factor inhibitor treatment [34]. Another study from the same Italian registry reported a 2-year retention rate of 47% for golimumab; however, similar retention rates between first- and second-line treatment were noted [31]. A recent retrospective study observed 3-year retention rates of 53% for infliximab and 76% for abatacept in biologic-naïve and -failure patients with RA [33]. In general, reported biologic retention rates are higher for patients who are treated earlier.

As previously reported with abatacept [10, 25, 26, 36] and other bDMARDs [37, 38], the clinical outcomes reported here were higher in biologic-naïve patients than in those with failure of prior biologic treatments and may suggest a greater benefit of earlier treatment [4]. The results from a study with tocilizumab showed equal efficacy in biologic-naïve patients as those with 1 or 2 previous bDMARD failures; however, overall treatment effectiveness was lower in those with > 2 failures [32]. Data from a Pan-European registry analysis also demonstrated that RF and ACPA positivity were associated with better abatacept retention [39].

Safety profiles for abatacept were similar across treatment lines in this study. The safety data reported were consistent with those previously reported for clinical trials [30] and real-life experience with SC abatacept [40], and with real-world IV abatacept studies (e.g. 8% of patients reported SAEs in ACTION) [24]. Similarly, the reported safety, efficacy, and retention rates observed for tocilizumab were comparable with IV and SC formulations [41, 42].

The SC route of administration is preferred by most patients due to its enhanced convenience and flexibility compared to IV administration [43]. The data reported here provide clinicians with valuable real-world evidence for the use of bDMARDs in a large population of international patients with RA in clinical settings.

The known inherent limitations of observational real-world studies include referral and channelling bias, the absence of an active comparator, and the loss of patients to follow-up. However, in the current study, few patients were lost to follow-up over 2 years (2.9%), which may be due in part to the study methodology not interfering with the usual clinical care of patients with RA. Conversely, as there was no study requirement for the investigator to perform follow-up visits or clinical assessments in addition to their routine clinical practices (consistent with other clinical practice studies worldwide), the data generated could be considered incomplete compared to data obtained from more stringently controlled clinical trials. Furthermore, due to the voluntary basis of the study, the participating rheumatologists may not be fully representative of all clinicians, although this potential bias is unavoidable and its impact is difficult to directly assess.

## Conclusions

In the ASCORE study, a 47% SC abatacept retention rate was observed at 2 years along with good clinical efficacy and safety outcomes in a real-world setting. Abatacept retention and clinical response rates were higher in patients who received abatacept earlier (compared with later) in their biologic drug treatment history and in those with RF/ACPA double-positive RA (compared with RF/ACPA double-negative RA). In routine clinical practice, overall retention of SC abatacept was similar to that of IV abatacept.

When determining the most appropriate treatment option for a patient, the route of administration can be an important factor for consideration; for example, SC formulations provide greater convenience as they can be administered by the patients at home [44]. These clinically applicable findings for SC abatacept have the potential to inform individualised treat-to-target plans for optimal and flexible therapeutic management of patients with moderate-to-severe RA.

## Supplementary Information

Below is the link to the electronic supplementary material.

Supplementary file1 (PDF 140 KB)

## Data Availability

The Bristol Myers Squibb policy on data sharing may be found at https://www.bms.com/researchers-and-partners/independent-research/data-sharing-request-process.html.

## References

[1] Combe B (2009). Progression in early rheumatoid arthritis. Best Pract Res Clin Rheumatol.

[2] Katchamart W, Koolvisoot A, Aromdee E, Chiowchanwesawakit P, Muengchan C (2015). Associations of rheumatoid factor and anti-citrullinated peptide antibody with disease progression and treatment outcomes in patients with rheumatoid arthritis. Rheumatol Int.

[3] Singh JA, Saag KG, Bridges SL, Akl EA, Bannuru RR, Sullivan MC, Vaysbrot E, McNaughton C, Osani M, Shmerling RH, Curtis JR, Furst DE, Parks D, Kavanaugh A, O'Dell J, King C, Leong A, Matteson EL, Schousboe JT, Drevlow B, Ginsberg S, Grober J, St Clair EW, Tindall E, Miller AS, McAlindon T (2016). 2015 American College of Rheumatology guideline for the treatment of rheumatoid arthritis. Arthritis Care Res.

[4] Smolen JS, Landewe RBM, Bijlsma JWJ, Burmester GR, Dougados M, Kerschbaumer A, McInnes IB, Sepriano A, van Vollenhoven RF, de Wit M, Aletaha D, Aringer M, Askling J, Balsa A, Boers M, den Broeder AA, Buch MH, Buttgereit F, Caporali R, Cardiel MH, De Cock D, Codreanu C, Cutolo M, Edwards CJ, van Eijk-Hustings Y, Emery P, Finckh A, Gossec L, Gottenberg JE, Hetland ML, Huizinga TWJ, Koloumas M, Li Z, Mariette X, Muller-Ladner U, Mysler EF, da Silva JAP, Poor G, Pope JE, Rubbert-Roth A, Ruyssen-Witrand A, Saag KG, Strangfeld A, Takeuchi T, Voshaar M, Westhovens R, van der Heijde D (2020). EULAR recommendations for the management of rheumatoid arthritis with synthetic and biological disease-modifying antirheumatic drugs: 2019 update. Ann Rheum Dis.

[5] Malmstrom V, Catrina AI, Klareskog L (2017). The immunopathogenesis of seropositive rheumatoid arthritis: from triggering to targeting. Nat Rev Immunol.

[6] Genovese MC, Covarrubias A, Leon G, Mysler E, Keiserman M, Valente R, Nash P, Simon-Campos JA, Porawska W, Box J, Legerton C, Nasonov E, Durez P, Aranda R, Pappu R, Delaet I, Teng J, Alten R (2011). Subcutaneous abatacept versus intravenous abatacept: a phase IIIb noninferiority study in patients with an inadequate response to methotrexate. Arthritis Rheumatol.

[7] Genovese MC, Schiff M, Luggen M, Becker JC, Aranda R, Teng J, Li T, Schmidely N, Le BM, Dougados M (2008). Efficacy and safety of the selective co-stimulation modulator abatacept following 2 years of treatment in patients with rheumatoid arthritis and an inadequate response to anti-tumour necrosis factor therapy. Ann Rheum Dis.

[8] Westhovens R, Kremer J, Emery P, Russell A, Alten R, Barre E, Dougados M (2014). Long-term safety and efficacy of abatacept in patients with rheumatoid arthritis and an inadequate response to methotrexate: a 7-year extended study. Clin Exp Rheumatol.

[9] Gottenberg JE, Ravaud P, Cantagrel A, Combe B, Flipo RM, Schaeverbeke T, Houvenagel E, Gaudin P, Loeuille D, Rist S, Dougados M, Sibilia J, Le Loet X, Marcelli C, Bardin T, Pane I, Baron G, Mariette X (2012). Positivity for anti-cyclic citrullinated peptide is associated with a better response to abatacept: data from the ‘Orencia and Rheumatoid Arthritis’ registry. Ann Rheum Dis.

[10] Alten R, Nüßlein H, Galeazzi M, Lorenz HM, Mariette X, Cantagrel A, Chartier M, Elbez Y, Rauch C, Le Bars M (2016). Do predictors of IV abatacept retention depend on the line of rheumatoid arthritis treatment: 12-month interim analysis of the observational, prospective ACTION study. Ann Rheum Dis.

[11] Yoshida K, Radner H, Kavanaugh A, Sung YK, Bae SC, Kishimoto M, Matsui K, Okada M, Tohma S, Weinblatt ME, Solomon DH (2013). Use of data from multiple registries in studying biologic discontinuation: challenges and opportunities. Clin Exp Rheumatol.

[12] Vashisht P, Sayles H, Cannella AC, Mikuls TR, Michaud K (2016). Generalizability of patients with rheumatoid arthritis in biologic agent clinical trials. Arthritis Care Res (Hoboken).

[13] Alten R, Mariette X, Buch M, Caporali R, Flipo R-M, Forster A, Nurmohamed M, Patel Y, Peichl P, Sanmarti R, Chartier M, Heitzmann J, Rauch C, Connolly S (2019) ASCORE, a 2-year, observational, prospective multicentre study of subcutaneous abatacept for the treatment of rheumatoid arthritis in routine clinical practice: 1-year interim analysis. Ann Rheum Dis 78(Suppl 2):1639. Abstract AB0361

[14] Alten R, Mariette X, Buch M, Caporali R, Flipo R-M, Forster A, Nurmohamed M, Patel Y, Peichl P, Sanmarti R, Chartier M, Heitzmann J, Rauch C, Connolly SE (2018) Experience with subcutaneous abatacept in routine clinical practice: 6-month interim analysis of a 2-year, prospective, non-interventional, multicentre study in patients with RA. Ann Rheum Dis 77(Suppl 2):1392. Abstract AB0461

[15] (1997) World Medical Association Declaration of Helsinki. Recommendations guiding physicians in biomedical research involving human subjects. JAMA 277:925–926 9062334

[16] (2001) ICH harmonised tripartite guideline guideline for good clinical practice. J Postgrad Med 47:199–203 11832625

[17] International Society for Pharmacoepidemiology (2016) Guidelines for good pharmacoepidemiology practices (GPP). 2020. https://www.pharmacoepi.org/resources/policies/guidelines-08027/. Accessed July 1, 2020

[18] Aletaha D, Neogi T, Silman AJ, Funovits J, Felson DT, Bingham CO 3rd, Birnbaum NS, Burmester GR, Bykerk VP, Cohen MD, Combe B, Costenbader KH, Dougados M, Emery P, Ferraccioli G, Hazes JM, Hobbs K, Huizinga TW, Kavanaugh A, Kay J, Kvien TK, Laing T, Mease P, Menard HA, Moreland LW, Naden RL, Pincus T, Smolen JS, Stanislawska-Biernat E, Symmons D, Tak PP, Upchurch KS, Vencovsky J, Wolfe F, Hawker G (2010) 2010 rheumatoid arthritis classification criteria: an American College of Rheumatology/European League Against Rheumatism collaborative initiative. Arthritis Rheum 62:2569–2581 10.1002/art.27584 20872595

[19] Felson DT, Smolen JS, Wells G, Zhang B, van Tuyl LH, Funovits J, Aletaha D, Allaart CF, Bathon J, Bombardieri S, Brooks P, Brown A, Matucci-Cerinic M, Choi H, Combe B, de Wit M, Dougados M, Emery P, Furst D, Gomez-Reino J, Hawker G, Keystone E, Khanna D, Kirwan J, Kvien TK, Landewe R, Listing J, Michaud K, Martin-Mola E, Montie P, Pincus T, Richards P, Siegel JN, Simon LS, Sokka T, Strand V, Tugwell P, Tyndall A, van der Heijde D, Verstappen S, White B, Wolfe F, Zink A, Boers M (2011). American College of Rheumatology/European League against Rheumatism provisional definition of remission in rheumatoid arthritis for clinical trials. Ann Rheum Dis.

[20] Liu P-C, Ssu C-T, Tsao Y-P, Liou T-L, Tsai C-Y, Chou C-T, Chen M-H, Leu C-M (2020). Cytotoxic T lymphocyte-associated antigen-4-Ig (CTLA-4-Ig) suppresses Staphylococcus aureus-induced CD80, CD86, and pro-inflammatory cytokine expression in human B cells. Arthritis Res Ther.

[21] Finckh A, Gomez-Reino J, Iannone F, Lie E, Canhao H, Pavelka K, Turesson C, Mariette X, Gottenberg JE, Hetland M, ABA oboP-E-Ro (2014) The impact of DMARD co-therapy on abatacept effectiveness in rheumatoid arthritis patients. A pan-European analysis of RA registries. Ann Rheum Dis 73(Suppl 2):492–493. Abstract FRI0298. 10.1136/annrheumdis-2014-eular.3004

[22] Finckh A, Neto D, Iannone F, Loza E, Lie E, van Riel P, Hetland ML, Pavelka K, Gottenberg JE, Canhão H, Mariette X, Turesson C (2015) The impact of patient heterogeneity and socio-economic factors on abatacept retention in rheumatoid arthritis across nine European countries. RMD Open 1:e000040 10.1136/rmdopen-2014-000040 PMC4613166 26509062

[23] Gottenberg JE, Morel J, Perrodeau E, Bardin T, Combe B, Dougados M, Flipo RM, Saraux A, Schaeverbeke T, Sibilia J, Soubrier M, Vittecoq O, Baron G, Constantin A, Ravaud P, Mariette X, French Society of Rheumatology and the investigators participating in AIR, ORA, and REGATE registries (2019). Comparative effectiveness of rituximab, abatacept, and tocilizumab in adults with rheumatoid arthritis and inadequate response to TNF inhibitors: prospective cohort study. BMJ.

[24] Alten R, Mariette X, Lorenz HM, Nusslein H, Galeazzi M, Navarro F, Chartier M, Heitzmann J, Poncet C, Rauch C, Le Bars M (2019). Predictors of abatacept retention over 2 years in patients with rheumatoid arthritis: results from the real-world ACTION study. Clin Rheumatol.

[25] Alten R, Mariette X, Lorenz HM, Galeazzi M, Cantagrel A, Nusslein HG, Chartier M, Elbez Y, Rauch C, Le Bars M (2017). Real-world predictors of 12-month intravenous abatacept retention in patients with rheumatoid arthritis in the ACTION observational study. RMD Open.

[26] Nüßlein HG, Alten R, Galeazzi M, Lorenz HM, Nurmohamed MT, Bensen WG, Burmester GR, Peter HH, Peichl P, Pavelka K, Chartier M, Poncet C, Rauch C, Le BM (2016). Efficacy and prognostic factors of treatment retention with intravenous abatacept for rheumatoid arthritis: 24-month results from an international, prospective, real-world study. Clin Exp Rheumatol.

[27] Salmon JH, Letarouilly JG, Goëb V, Kanagaratnam L, Coquerelle P, Guyot MH, Houvenagel E, Lecuyer N, Marguerie L, Morel G, Baudens G, Gervais E, Flipo RM (2020). Actual persistence of abatacept in rheumatoid arthritis: results of the French-Ric network. J Clin Med.

[28] Genovese MC, Pacheco-Tena C, Covarrubias A, Leon G, Mysler E, Keiserman M, Valente R, Nash P, Simon-Campos JA, Box J, Legerton CW, Nasonov E, Durez P, Delaet I, Teng J, Alten R (2014). Subcutaneous abatacept for the treatment of rheumatoid arthritis: longterm data from the ACQUIRE trial. J Rheumatol.

[29] Schiff M, Keiserman M, Codding C, Songcharoen S, Berman A, Nayiager S, Saldate C, Aranda R, Becker JC, Nys M, Le BM, Reed DM, Poncet C, Dougados M (2011). Clinical response and tolerability to abatacept in patients with rheumatoid arthritis previously treated with infliximab or abatacept: open-label extension of the ATTEST Study. Ann Rheum Dis.

[30] Schiff M, Weinblatt ME, Valente R, van der Heijde D, Citera G, Elegbe A, Maldonado M, Fleischmann R (2014). Head-to-head comparison of subcutaneous abatacept versus adalimumab for rheumatoid arthritis: two-year efficacy and safety findings from AMPLE trial. Ann Rheum Dis.

[31] Manara M, Caporali R, Favalli EG, Grosso V, Atzeni F, Sarzi Puttini P, Gorla R, Bazzani C, Fusaro E, Pellerito R, Rocchetta PA, Sinigaglia L (2017). Two-year retention rate of golimumab in rheumatoid arthritis, psoriatic arthritis and ankylosing spondylitis: data from the LORHEN registry. Clin Exp Rheumatol.

[32] Baganz L, Richter A, Kekow J, Bussmann A, Krause A, Stille C, Listing J, Zink A, Strangfeld A (2018). Long-term effectiveness of tocilizumab in patients with rheumatoid arthritis, stratified by number of previous treatment failures with biologic agents: results from the German RABBIT cohort. Rheumatol Int.

[33] Ebina K, Hashimoto M, Yamamoto W, Hirano T, Hara R, Katayama M, Onishi A, Nagai K, Son Y, Amuro H, Yamamoto K, Maeda Y, Murata K, Jinno S, Takeuchi T, Hirao M, Kumanogoh A, Yoshikawa H (2019). Drug tolerability and reasons for discontinuation of seven biologics in 4466 treatment courses of rheumatoid arthritis—the ANSWER cohort study. Arthritis Res Ther.

[34] Favalli EG, Sinigaglia L, Becciolini A, Grosso V, Gorla R, Bazzani C, Atzeni F, Sarzi Puttini PC, Fusaro E, Pellerito R, Caporali R (2018). Two-year persistence of golimumab as second-line biologic agent in rheumatoid arthritis as compared to other subcutaneous tumor necrosis factor inhibitors: real-life data from the LORHEN registry. Int J Rheum Dis.

[35] Notario Ferreira I, Ferrer Gonzalez MA, Morales Garrido P, Gonzalez Utrilla A, Garcia Sanchez A, Soto Pino MJ, Suero Rosario E, Caro Hernandez C, Anon Onate I, Perez Albaladejo L, Caliz Caliz R (2017). Two-year efficacy of tocilizumab in patients with active rheumatoid arthritis in clinical practice. Reumatol Clin.

[36] Ogawa N, Ohashi H, Ota Y, Kobori K, Suzuki M, Tsuboi S, Hayakawa M, Goto Y, Karahashi T, Kimoto O, Miyamoto T, Furukawa S, Shimoyama K, Suzuki D, Maekawa Y (2019). Multicenter, observational clinical study of abatacept in Japanese patients with rheumatoid arthritis. Immunol Med.

[37] Mori S, Yoshitama T, Ueki Y (2018). Tofacitinib therapy for rheumatoid arthritis: a direct comparison study between biologic-naive and experienced patients. Intern Med.

[38] Greenberg JD, Reed G, Decktor D, Harrold L, Furst D, Gibofsky A, Dehoratius R, Kishimoto M, Kremer JM (2012). A comparative effectiveness study of adalimumab, etanercept and infliximab in biologically naive and switched rheumatoid arthritis patients: results from the US CORRONA registry. Ann Rheum Dis.

[39] Gottenberg JE, Neto D, Gomez-Reino J, Iannone F, Lie E, Canhao H, Pavelka K, Turesson C, Hetland M, Mariette X, Finckh A (2014) Positivity for rheumatoid factor and anti-cyclic citrullinated peptide is associated with better drug retention of abatacept: data from a pan-European analysis of RA registries. Ann Rheum Dis 73 (Suppl 2):502. Abstract FRI0322

[40] Sarmiento-Monroy JC, Parada-Arias L, Rodriguez-Lopez M, Rodriguez-Jimenez M, Molano-Gonzalez N, Rojas-Villarraga A, Mantilla RD (2019). Subcutaneous abatacept in rheumatoid arthritis: a real-life experience. J Transl Autoimmun.

[41] Lauper K, Mongin D, Iannone F, Klami Kristianslund E, Kvien TK, Nordstrom D, Pavelka K, Pombo-Suarez M, Rotar Z, Santos MJ, Codreanu C, Lukina G, Courvoisier DS, Gabay C (2018). Comparative effectiveness of subcutaneous tocilizumab versus intravenous tocilizumab in a pan-European collaboration of registries. RMD Open.

[42] Burmester GR, Rubbert-Roth A, Cantagrel A, Hall S, Leszczynski P, Feldman D, Rangaraj MJ, Roane G, Ludivico C, Bao M, Rowell L, Davies C, Mysler EF (2016). Efficacy and safety of subcutaneous tocilizumab versus intravenous tocilizumab in combination with traditional DMARDs in patients with RA at week 97 (SUMMACTA). Ann Rheum Dis.

[43] Desplats M, Pascart T, Jelin G, Norberciak L, Philippe P, Houvenagel E, Goeb V, Flipo RM (2017). Are abatacept and tocilizumab intravenous users willing to switch for the subcutaneous route of administration? A questionnaire-based study. Clin Rheumatol.

[44] Wells AF, Jodat N, Schiff M (2014). A critical evaluation of the role of subcutaneous abatacept in the treatment of rheumatoid arthritis: patient considerations. Biologics.

