# Supplementary material for: Retention of subcutaneous abatacept for the treatment of rheumatoid arthritis: real-world results from the ASCORE study: an international 2-year observational study
Source: Clin Rheumatol. 2022 May 10;41(8):2361–73. doi: 10.1007/s10067-022-06176-1 (PMC9287226; doi:10.1007/s10067-022-06176-1)

SUPPLEMENTARY INFORMATION

Retention of subcutaneous abatacept for the treatment of rheumatoid arthritis: real-world results from the ASCORE study: an international 2-year observational study

Rieke Alten, Xavier Mariette, René-Marc Flipo, Roberto Caporali, Maya H Buch, Yusuf Patel, Sara Marsal, Raimon Sanmartí, Michael T Nurmohamed, Hedley Griffiths, Peter Peichl, Bettina Bannert, Melanie Chartier, Sean E Connolly, Karissa Lozenski, Christiane Rauch

Correspondence:

Rieke Alten

Schlosspark-Klinik,

Email: Rieke.alten@schlosspark-klinik.de

Supplemental table 1 Summary of enrolment in the ASCORE study by country

Country	Rheumatologists N=574	Patients enrolled N=2945[a]
Australia	14	78
Austria	16	61
France[b]	157	553
Germany	151	901
Greece	19	128
Italy	73	484
Netherlands	21	124
Spain	64	278
Switzerland	12	30
UK	47	308

[a]N=2956; 11 patients with no recorded date of consent or date of visit 1 were excluded.

[b]Including Monaco.

Supplemental table 2 Baseline patient demographics and disease characteristics for patients with RF/ACPA serostatus data (n=1748)

	**+/+ RA (n=1079)**		**+/− RA (n=326)**		**−/− RA (n=343)**	
	**Biologic-naïve (n=511)**	**≥2 prior biologics (n=568)**	**Biologic-naïve (n=140)**	**≥2 prior biologics (n=186)**	**Biologic-naïve (n=140)**	**≥2 prior biologics (n=203)**
Age, years	57.1 (13.4)	57.1 (12.2)	57.0 (12.6)	58.4 (13.0)	59.5 (14.7)	56.6 (13.2)
Disease duration, years	8.4 (8.7)	13.2 (9.5)	6.9 (7.0)	13.4 (9.7)	7.1 (7.9)	10.3 (7.7)
DAS28 (CRP)	4.7 (1.2)	4.7 (1.2)	4.4 (1.1)	4.5 (1.0)	4.8 (1.1)	4.8 (1.2)
CDAI	26.6 (12.5)	26.6 (12.4)	24.1 (12.8)	25.0 (10.3)	27.7 (12.5)	28.6 (13.8)
SDAI	28.1 (13.1)	28.1 (12.9)	24.7 (12.4)	26.5 (11.0)	29.1 (12.9)	30.2 (14.7)

Data are shown as mean (SD). Patients with missing data for baseline RF/ACPA status are excluded. Reproduced from Alten R, *et al*. EULAR Virtual Congress 2021; 3 June 2021; oral presentation OP0180 (with permission from the authors).

ACPA, anti-citrullinated protein antibody; CRP, C-reactive protein; DAS28, Disease Activity Score in 28 joints; RA, rheumatoid arthritis; RF, rheumatoid factor; SD, standard deviation.

Supplemental table 3 Summary of retention rates of other biologics reported in literature

Study type	Length, years	Treatment	Population	Retention, %
Clinical practice [35]	2	TCZ	All patients	61.0
Registry (RABBIT) [32]	3	TCZ	bDMARD naïve<br>1 prior bDMARD failure<br>2 prior bDMARD failures	52.2<br>50.8<br>46.5
Registry (AIR and REGATE) [23]	2	RTX<br>TCZ	Patients had a median of 2 prior TNFi failures	68.6<br>63.4
Registry (LORHEN) [34]	2	ADA<br>ETN<br>GLM	Previous TNFi failure	31.2<br>39.8<br>53.4
Registry (LORHEN) [31]	2	GLM	All patients	47.3
Retrospective (ANSWER) [33]	3	IFX<br>GLM<br>TCZ<br>ETN<br>CZP<br>ADA	Biologic naïve and biologic failure	53.4<br>65.6<br>71.5<br>61.2<br>60.7<br>58.2

ADA, adalimumab; bDMARD, biologic disease-modifying antirheumatic drug; CZP, certolizumab pegol; ETN, etanercept; GLM, golimumab; IFX, infliximab; RTX, rituximab; TCZ, tocilizumab; TNFi, tumour necrosis factor inhibitor.

Supplemental figure 1 Exploratory analysis including patients who discontinued SC and switched to the IV formulation of abatacept.

CI, confidence interval; IV, intravenous; SC, subcutaneous.

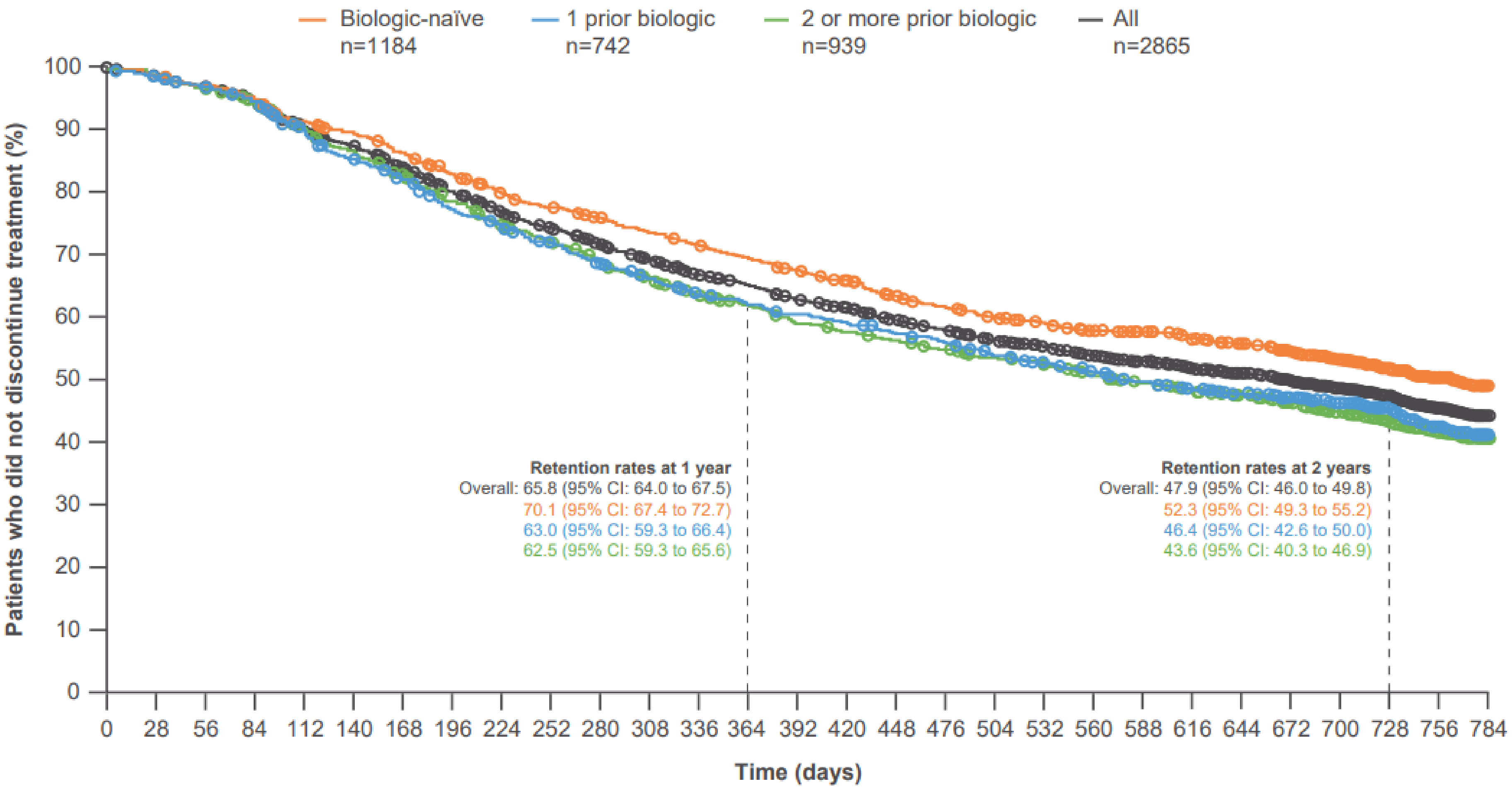

Supplement: Supplementary file 1 — Supplementary file1 (PDF 140 KB) [file 10067_2022_6176_MOESM1_ESM.pdf]
